# Saúde Cardiovascular Feminina: Um Desafio Atual

**DOI:** 10.36660/abc.20260532

**Published:** 2026-09-08

**Affiliations:** 1 Universidade Federal do Rio de Janeiro Rio de Janeiro RJ Brasil Universidade Federal do Rio de Janeiro, Rio de Janeiro, RJ – Brasil; 2 Clínica São Vicente Rio de Janeiro RJ Brasil Clínica São Vicente, Rio de Janeiro, RJ – Brasil; 3 Hospital Quinta D'Or Rio de Janeiro RJ Brasil Hospital Quinta D'Or, Rio de Janeiro, RJ – Brasil

**Keywords:** Saúde da Mulher, Mortalidade, Epidemiologia, América Latina, Região do Caribe

A crescente atenção dedicada à saúde cardiovascular da mulher reflete uma mudança de paradigma necessária na medicina contemporânea. Essa transformação fundamenta-se na evidência de que as doenças cardiovasculares (DCV) constituem a principal causa de morte entre as mulheres, sendo responsáveis por aproximadamente 35% do total de óbitos femininos globais, superando a mortalidade por todas as neoplasias combinadas.^1^

Apesar da expansão das campanhas de conscientização e dos avanços no conhecimento científico sobre o tema, observa-se uma preocupante estagnação na redução da carga global dessas doenças na última década. As mulheres ainda enfrentam menor reconhecimento do seu risco cardiovascular, atraso no diagnóstico e menor acesso a terapias baseadas em evidências.^1,2^ Na América Latina e no Caribe (ALC), esse cenário é ainda mais desafiador, pois a presença de desigualdades sociais e estruturais, impõe barreiras adicionais ao diagnóstico e ao tratamento adequado das DCV.^3,4^

Sob o ponto de vista epidemiológico, pode-se entender a magnitude das DCV em mulheres como resultado da interação entre fatores de risco (FR) tradicionais, biológicos e emergentes. Na ALC, a hipertensão arterial (HA) sistólica representa o principal FR atribuível à mortalidade cardiovascular feminina, seguida pela dislipidemia e pelo diabetes mellitus (DM).^4^ Além disso, tabagismo e DM conferem risco relativo de doença arterial coronariana (DAC) proporcionalmente maior em mulheres do que em homens.^4^ Somam-se a estes, os FR específicos da mulher, como as doenças hipertensivas da gestação, diabetes gestacional, síndrome dos ovários policísticos e menopausa precoce, que constituem importantes marcadores de risco cardiovascular ao longo da vida e contribuem para o envelhecimento cardiovascular precoce.^5,6^

Os fatores emergentes como depressão, violência doméstica, sobrecarga de papéis sociais e privação socioeconômica, por sua vez, são particularmente prevalentes na ALC e exercem influência relevante sobre o risco cardiovascular feminino de forma sinérgica, contribuindo para a inflamação sistêmica e disfunção endotelial. A interação entre esses múltiplos FR reforça que a saúde cardiovascular da mulher latino-americana e caribenha é influenciada de maneira importante pelo ambiente e pelos determinantes sociais da saúde.^7^

Adicionalmente, as disparidades regionais e socioeconômicas são pronunciadas na ALC quando analisamos as doenças cardiovasculares no sexo feminino. A prevalência e a mortalidade por estas doenças seguem um gradiente socioeconômico, com maior carga de morbimortalidade concentrada em populações socialmente vulneráveis.^3,8^ Mulheres com menor escolaridade e acesso limitado aos serviços de saúde apresentam maior prevalência de HA e DM, além de maior comprometimento funcional decorrente dessas condições.^7^ Embora a cardiopatia isquêmica permaneça como a principal causa de morte cardiovascular na região, doenças negligenciadas, como a cardiopatia reumática e a doença de Chagas, continuam exercendo uma carga desproporcional de morbimortalidade, especialmente entre mulheres jovens em idade reprodutiva.^1^

Nesse contexto, o estudo de Mesquita et al., publicado nesta edição do Arquivos Brasileiros de Cardiologia,^9^ traz importantes observações sobre as DCV na população feminina na ALC. Os resultados endossam que as DCV permanecem como a principal causa de morte entre as mulheres da região e demonstram maior prevalência de sedentarismo, dislipidemia, glicemia de jejum elevada e sobrepeso/obesidade em comparação aos homens. Além disso, observou-se a elevada frequência dos FR específicos do sexo feminino que, influenciados por determinantes sociais e econômicos, nos levam à reflexão sobre a adoção de estratégias de prevenção que considerem essas particularidades.^9^

Em relação ao impacto da pandemia de COVID-19 na saúde cardiovascular feminina, estudos demonstram exacerbação das disparidades de gênero pré-existentes,^10^ e entre as mulheres, maior impacto em mulheres hispânicas.^11^ No presente estudo, Mesquita et al. observaram que a COVID-19 foi a principal causa de morte nas Américas em 2021 e, além de ter sobrecarregado os sistemas de saúde e comprometido estratégias de prevenção, evidenciou o impacto dos FR cardiometabólicos nas mulheres.^9^

De maneira semelhante, em relação à mortalidade prematura por DCV, os autores também observaram desigualdades entre as mulheres, o que pode estar relacionado à indicadores sociais da saúde.^9^ O contraste entre Haiti e Guiana, com as maiores probabilidades de óbito entre 30 e 70 anos, e o Canadá, com a menor mortalidade prematura, ilustra o impacto da prevenção, do acesso aos serviços de saúde e do controle dos FR sobre a sobrevida feminina.^9^

Desta forma, observamos que apesar dos avanços no reconhecimento das lacunas da saúde cardiovascular da mulher, importantes barreiras ainda existem para a melhoria dos desfechos clínicos. Mulheres continuam menos propensas a receber terapias baseadas em evidências e intervenções oportunas para DCV agudas,^3,12^ os escores tradicionais frequentemente subestimam seu risco por não incorporarem fatores específicos do sexo,^1,13^ e há persistente sub-representação feminina em ensaios clínicos, particularmente de mulheres latino-americanas, negras e gestantes, limitando a produção de evidências capazes de orientar uma assistência verdadeiramente equitativa.^7^

Por isso, superar esse cenário exige estratégias integradas que transcendem o cuidado clínico. O fortalecimento de políticas públicas voltadas aos determinantes sociais da saúde, a incorporação sistemática dos FR específicos do sexo feminino na prática clínica, a expansão de programas de cardio-obstetrícia, a ampliação do acesso por meio de tecnologias digitais e uma maior representatividade feminina na pesquisa científica constituem importantes tópicos para uma abordagem mais equitativa e esclarecedora do tema. Assim, modificar a forma sobre como a saúde cardiovascular da mulher é compreendida, investigada e incorporada às políticas de saúde deve ser parte fundamental da estratégia global para a redução de desfechos clínicos.
