# Supplementary material for: Saúde Cardiovascular Feminina: Um Desafio Atual
Source: Arq Bras Cardiol. 2026 Sep 8;123(7):e20260532. [Article in Portuguese] doi: 10.36660/abc.20260532 (PMC13668457; doi:10.36660/abc.20260532)
Supplement: Translation

# Translation: Women's Cardiovascular Health: A Current Challenge

**DOI:** 10.36660/abc.20260532i

**Affiliations:** 1 Rio de Janeiro RJ Brazil Universidade Federal do Rio de Janeiro, Rio de Janeiro, RJ – Brazil; 2 Rio de Janeiro RJ Brazil Clínica São Vicente, Rio de Janeiro, RJ – Brazil; 3 Rio de Janeiro RJ Brazil Hospital Quinta D'Or, Rio de Janeiro, RJ – Brazil

**Keywords:** Women's Health, Mortality, Epidemiology, Latin America, Caribbean Region

The growing attention paid to women's cardiovascular health reflects a necessary paradigm shift in contemporary medicine. This transformation is based on evidence that cardiovascular diseases (CVD) are the leading cause of death among women, accounting for approximately 35% of all global female deaths, surpassing mortality from all cancers combined.^1^

Despite the expansion of awareness campaigns and advances in scientific knowledge on the subject, a worrying stagnation in the reduction of the global burden of these diseases has been observed in the last decade. Women still face less recognition of their cardiovascular risk, delays in diagnosis, and less access to evidence-based therapies.^1,2^ In Latin America and the Caribbean (LAC), this scenario is even more challenging, as the presence of social and structural inequalities imposes additional barriers to the diagnosis and adequate treatment of CVD.^3,4^

From an epidemiological standpoint, the magnitude of CVD in women can be understood as a result of the interaction between traditional, biological, and emerging risk factors (RF). In LAC, systolic hypertension (SH) represents the main RF attributable to female cardiovascular mortality, followed by dyslipidemia and diabetes mellitus (DM).^4^ Furthermore, smoking and DM confer a proportionally higher relative risk of coronary artery disease (CAD) in women than in men.^4^ Added to these are women-specific RF, such as hypertensive disorders of pregnancy, gestational diabetes, polycystic ovary syndrome, and early menopause, which constitute important markers of cardiovascular risk throughout life and contribute to premature cardiovascular aging.^5,6^

Emerging factors such as depression, domestic violence, overload of social roles, and socioeconomic deprivation, in turn, are particularly prevalent in LAC and exert a significant influence on women's cardiovascular risk in a synergistic way, contributing to systemic inflammation and endothelial dysfunction. The interaction between these multiple RF reinforces that the cardiovascular health of Latin American and Caribbean women is significantly influenced by the environment and the social determinants of health.^7^

Additionally, regional and socioeconomic disparities are pronounced in LAC when analyzing cardiovascular diseases in women. The prevalence and mortality from these diseases follow a socioeconomic gradient, with a higher burden of morbidity and mortality concentrated in socially vulnerable populations.^3,8^ Women with lower education levels and limited access to health services have a higher prevalence of hypertension and diabetes mellitus, as well as greater functional impairment resulting from these conditions.^7^ Although ischemic heart disease remains the leading cause of cardiovascular death in the region, neglected diseases such as rheumatic heart disease and Chagas disease continue to exert a disproportionate burden of morbidity and mortality, especially among young women of reproductive age.^1^

In this context, the study by Mesquita et al., published in this issue of *Arquivos Brasileiros de Cardiologia*,^9^ provides important observations on CVD in the female population in LAC. The results confirm that CVD remains the leading cause of death among women in the region and demonstrate a higher prevalence of sedentary lifestyle, dyslipidemia, elevated fasting glucose, and overweight/obesity compared to men. Furthermore, a high frequency of female-specific RF was observed, which, influenced by social and economic determinants, leads us to reflect on the adoption of prevention strategies that consider these particularities.^9^

Regarding the impact of the COVID-19 pandemic on women's cardiovascular health, studies demonstrate an exacerbation of pre-existing gender disparities,^10^ and among women, with a greater impact on Hispanic women.^11^ In the present study, Mesquita et al. observed that COVID-19 was the leading cause of death in the Americas in 2021 and, in addition to overburdening health systems and compromising prevention strategies, highlighted the impact of cardiometabolic RF in women.^9^

Similarly, regarding premature mortality from CVD, the authors also observed inequalities among women, which may be related to social indicators of health. ^9^ The contrast between Haiti and Guyana, with the highest probabilities of death between 30 and 70 years of age, and Canada, with the lowest premature mortality, illustrates the impact of prevention, access to health services, and control of risk factors on female survival.^9^

Thus, we observe that despite advances in recognizing gaps in women's cardiovascular health, significant barriers still exist to improving clinical outcomes. Women remain less likely to receive evidence-based therapies and timely interventions for acute CVD,^3,12^ traditional scoring systems often underestimate risk because they do not incorporate sex-specific factors,^1,13^ and there is persistent underrepresentation of women in clinical trials, particularly Latin American, black, and pregnant women, limiting the production of evidence capable of guiding truly equitable care.^7^

Therefore, overcoming this scenario requires integrated strategies that transcend clinical care. Strengthening public policies focused on the social determinants of health, the systematic incorporation of female-specific RF into clinical practice, the expansion of cardio-obstetrics programs, increased access through digital technologies, and greater female representation in scientific research are important topics for a more equitable and enlightening approach to the subject. Thus, changing how women's cardiovascular health is understood, investigated, and incorporated into health policies should be a fundamental part of the overall strategy for reducing clinical outcomes.
